# Multigenerational mistimed feeding drives circadian reprogramming with an impaired unfolded protein response

**DOI:** 10.3389/fendo.2023.1157165

**Published:** 2023-03-06

**Authors:** Kai Huang, Tao Zhang, Wenjun Zhang, Yue Gu, Pan Yu, Lanqing Sun, Zhiwei Liu, Tao Wang, Ying Xu

**Affiliations:** ^1^ Jiangsu Key Laboratory of Neuropsychiatric Diseases and Cambridge-Su Genomic Resource Center, Medical School of Soochow University, Suzhou, Jiangsu, China; ^2^ Department of Laboratory Medicine, Affiliated Hospital of Jiangnan University, Wuxi, Jiangsu, China

**Keywords:** mistimed feeding, multigenerational effect, rhythm reprogramming, unfolded protein response, ER stress

## Abstract

Mistimed food intake in relation to the day/night cycle disrupts the synchrony of circadian rhythms in peripheral tissues and increases the risk of metabolic diseases. However, the health effects over generations have seldom been explored. Here, we established a 10-generation mouse model that was continuously fed with daytime-restricted feeding (DRF). We performed RNA-seq analysis of mouse liver samples obtained every 4 h over a 24 h period from F2, F5 and F10 generations exposed to DRF. Multigenerational DRF programs the diurnal rhythmic transcriptome through a gain or loss of diurnal rhythmicity over generations. Gene ontology (GO) analysis of the differential rhythmic transcriptome revealed that adaptation to persistent DRF is accompanied by impaired endoplasmic reticulum (ER) stress. Consistently, a substantially higher level of folding-deficient proinsulin was observed in F10 liver tissues than in F2 and F5 liver tissues following tail vein injection. Subsequently, tunicamycin induced more hepatocyte death in F10 samples than in F2 and F5 samples. These data demonstrate that mistimed food intake could produce cumulative effects over generations on ER stress sensitivity in mice.

## Introduction

Lifestyle has become more irregular in industrialized nations, including night shift work, which negatively affects our overall health and wellbeing. Irregular food intake itself may drive downstream dysregulation of circadian rhythms in peripheral tissues such as the liver and is believed to contribute to the development of numerous diseases (1). Furthermore, skipping breakfast or postponing dinner can lead to an increased risk of obesity (2–4). Most animals consume their food during their active phase of a daily cycle, which is the light phase for diurnal species and the dark phase for nocturnal species. Daytime-restricted feeding (DRF) programs glucose intolerance and impairs insulin secretion in nocturnal rat offspring (5). Metabolic organs receive food at the wrong time with subsequent temporal disruption of anabolic and catabolic processes. These findings indicate that individuals who eat at the wrong time may have progeny with hepatic stress and dysfunction.

The circadian system is a hierarchically organized multi-oscillator network consisting of a central clock located in the suprachiasmatic nucleus (SCN) and oscillators in peripheral organs (6, 7). The SCN coordinates peripheral oscillators to align local clocks with geophysical time (8). These clocks are responsive to environmental cues (e.g., light, food, temperature) to accommodate daily recurring environmental changes by altering clock gene expression or protein levels (9–11). Although the master clock SCN is sensitive to light, feeding and fasting are the dominant environmental cues for many other tissues, such as the liver (12). Short-term studies of DRF in mice showed a 12 h phase shift of the expression of liver and white adipose tissue (WAT) clock genes (13). In liver and visceral adipose tissue (VAT), 61% and 80.5% rhythmic genes reach the antiphase state after a 7-day DRF, which are enriched in fatty acid metabolism, complex carbohydrates metabolism, transport of lipids, and glucose metabolism (14). Although changes in rhythmic gene expression caused by out-of-phase feeding are well described in mice, it is not known whether inverted meal timing induces a cumulative impact on rhythmic gene expression over generations.

Endoplasmic reticulum (ER) is an important organelle that serves many functions, particularly in protein synthesis, folding, and transport. ER stress signaling and the unfolded protein response (UPR) are triggered to restore cellular homeostasis, but a prolonged ER stress response can activate apoptotic signals, leading to damage to the target cells. External influences, such as nutrient supply, can disrupt the function and homeostasis of the ER, contributing to the progression of metabolic diseases (15). However, food intake with an improper dietary time and nutrient deprivation also disrupts ER homeostasis, representing an additional source of ER stress. Indeed, historical nutrition challenges influence the health and disease risk of offspring (16). Therefore, the question remains whether ER stress dysfunction in response to inverted meal timing could show an incremental impact on further generations.

In this study, we identified two clusters of rhythmic liver RNA expression that display a tendency to gain or lose rhythmicity from F2 to F10 in response to multigenerational DRF. We focused on the ER stress pathway, including the rhythmic changes in ER stress-associated genes and unfolded protein-mediated apoptotic cell death.

## Materials and methods

### Animals

All C57BL/6 mice were housed in a specific pathogen-free animal facility and maintained on a 12 h light:12 h dark cycle (lights on at 8 a.m.). F1 mice were derived from *ad libitum*-fed (Ad) mice and assigned to DRF from weaning. Mice of the following generations were under DRF for life until they were euthanized for experiments. DRF mice had access to food for 6 h from ZT2 to ZT8 (ZT = Zeitgeber time, ZT0 is light onset, ZT12 is light off).

### Body weight, food intake, metabolic rhythm measurement, and sperm count

Body weights were measured at birth and every 5 days from weaning until 2 months of age. Then, mice were placed into individual cages to record daily food intake. Consecutive mouse activity, food consumption and respiratory exchange ratio (RER) were measured by a comprehensive animal monitoring system (Oxymax; Columbus Instruments). Sperm was collected from epididymis as previously reported and calculated by MAKLER COUNTING CHAMBER (17).

### Triglyceride measurements

Blood was collected from the orbital sinus into sterile 1.5 ml tubes containing citrate sodium (3 M). Then, blood cells were removed by centrifugation at 2,000 g for 20 min at 4°C, the supernatant was immediately aliquoted and stored at -80°C. Plasma was prepared for TG analysis with 7100 automatic biochemical analyzer (Hitachi). Hepatic TG was performed according to the protocol of the TG assay kit (Nanjing Jiancheng Bioengineering Institute, A110-1-1)

### Tunicamycin administration

For the ER stress model, mice were intraperitoneally injected with tunicamycin (APExBIO) dissolved in dimethyl sulfoxide (DMSO) and diluted in sterile 150 mM dextrose at a dose of 0.5 μg/g body mass at ZT8, and tissues were sampled 24 h later.

### Hydrodynamic injection

Mice were anesthetized, and then, plasmid DNA suspended in sterile PBS in a volume equal to 10% of the body weight was injected in 5 to 7 s *via* the tail vein of mice. The amount of injected plasmid was 6 μg flag-tagged folding-deficient mutant proinsulin, and tissue samples were collected 20 h after injection.

### RNA sequencing

Liver samples were collected at 4 h intervals from ZT0 to ZT20 and immediately stored at -80°C. Total RNA was then extracted by the TRIzol procedure (Thermo Fisher). RNA purity and concentration were assessed by RNA electrophoresis, NanoDrop, and Agilent 2200 TapeStation analysis. RNA-seq was performed on an Illumina MiSeq platform with PE 150 bp reads at the BGI Genome Center, Shenzhen, China.

Raw sequence files were subjected to quality control analysis using FastQC. Reads were mapped to the mouse reference genome GRCm38/mm10 using bowtie (v1.1.2), and expression quantification was performed using RSEM (v1.2.8). The expression values were normalized by FPKM.

### Rhythmicity assessment and functional enrichment analysis

To assess rhythmicity in gene expression, we used the meta2d algorithm, a function of the MetaCycle *R* package (v1.2.0) (18). *P*<0.05 was considered significant. Functional enrichment analysis of circadian reprogramming genes was performed *via* Metascape (19).

### Quantitative real-time PCR

Total RNA from liver tissues was extracted using TRIzol reagent according to the manufacturer’s instructions. RNA (1 μg) was reverse transcribed using a PrimeScript RT Reagent Kit (TaKaRa). Quantitative real-time PCR was performed with SYBR Green detection reagent (TaKaRa). Measured values from specific genes were analyzed by the ΔCt method normalized to *Actin* as an endogenous control. Primer sequences for quantitative real-time PCR are listed in **Table S1**.

### Western blotting

Protein lysates from isolated tissues were extracted using RIPA buffer with protease inhibitors. The protein concentration was measured with a BCA assay, and an equal amount of protein was separated by SDS PAGE electrophoresis and transferred to PVDF membranes. Membranes were blocked with 5% milk powder in Tris-buffered saline-tween 20 (TBST) for 1 h and then incubated with specific primary antibody (in blocking solution) at 4°C overnight. Next, the membranes were incubated with horseradish peroxidase (HRP)-conjugated secondary antibodies for 1 h at RT. After several washes with TBST, the membrane was incubated with Omni ECL reagent (EpiZyme) and imaged by a ChemiScope 6200 system (CLINX). The following primary antibodies were used for the experiments. Antibodies against β-actin (1:5,000, A5441; Sigma-Aldrich), β-Tubulin (1:5,000, #5346; Cell Signaling), CHOP (1:1,000, #2895; Cell Signaling), Procaspase-3 (1:1,000, #9662; Cell Signaling), Cleaved Caspase-3 (1:1,000, #9664; Cell Signaling), BAX (1:1,000, #2772; Cell Signaling), and BCL2 (1:1,000, #3498; Cell Signaling) were used.

### Immunofluorescence assay

Fresh liver tissues were rapidly frozen in liquid nitrogen and mounted in OCT compound. Then, thin sections (6 μm) were cut, mounted onto poly-L-lysine-coated glass slides, fixed in 4% paraformaldehyde in PBS for 20 min at RT, and washed in PBS. Terminal transferase-mediated dUTP nick-end labeling (TUNEL) staining was performed according to the protocol of the (TUNEL) BrightRed Apoptosis Detection Kit (Vazyme, A113-01).

### Quantification and statistical analysis

All data are shown as the mean ± standard deviation (SD) unless otherwise specified. The results were evaluated using unpaired (two-tailed) Student’s *t* test and one- or two-way analysis of variance (ANOVA). *P*<0.05 was considered statistically significant (*P<0.05, **P<0.01, and ***P<0.001). All statistical analysis were performed using GraphPad Prism 9 (GraphPad Software, Inc., San Diego, CA, USA).

## Results

### Altered traits of mice with multigenerational DRF

**Figure 1:**
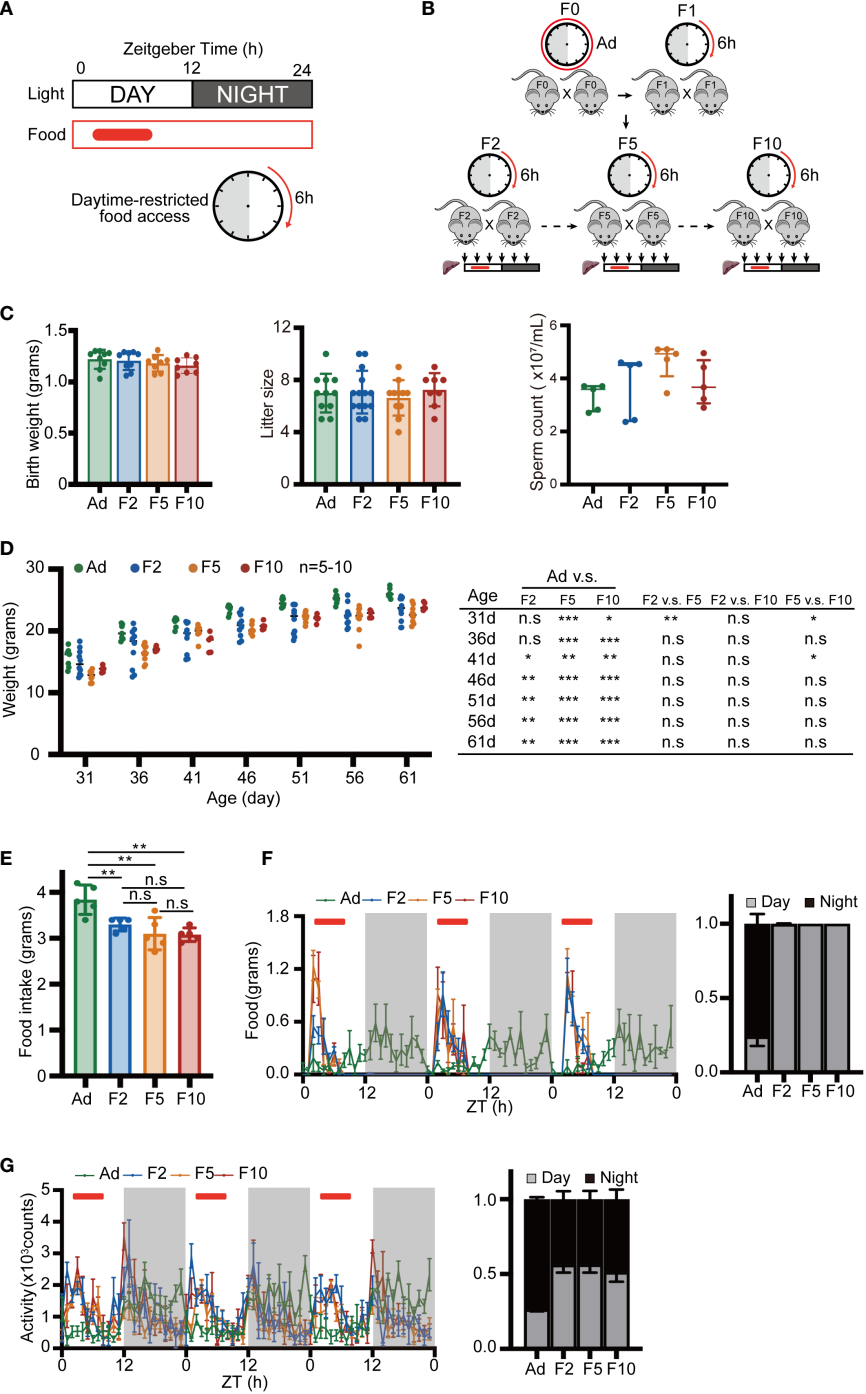
Basic behavior and metabolic measurements. **(A)** Schematic showing the DRF schedules. Day and night are indicated above with white and gray bars. The solid red box and the clock pattern indicate the timing of food access. **(B)** Schematic diagram of the multigenerational DRF mouse model. The sampling time points are indicated by black arrows. **(C)** Body weight of neonatal mice (n = 8-9), litter size (n = 8-14) and sperm number of adult mice (n = 5). **(D)** Body weight recorded at 5-day intervals from 31 days to 61 days (n= 5-10) and *P* value from statistical hypothesis test. **(E)** Daily food intake (n = 5). **(F, G)** Activity and food consumption recorded at 20 min intervals for 3 days; red bars indicate the timing of food access (Ad group were fed ad libitum). Histograms show the daily percentage of activity and food consumption; day and night are indicated with gray and black bars (n = 4). Significance was calculated by unpaired two-tailed Student’s *t* test, or two-way ANOVA; *P < 0.05; **P < 0.01; ***P < 0.001 were considered significant; n.s., not significant. Error bars represent the mean ± SD.

To illustrate the contribution of multigenerational DRF to physiology and rhythmic gene expression, we provided a normal chow diet to mice only in the inactive period (ZT2 to ZT8; ZT = Zeitgeber time, ZT0 is light onset, ZT12 is light off) (**Figure 1A**). F0 mice were derived from *ad libitum* mice and assigned to DRF after weaning. The offspring were fed from ZT2 to ZT8 until they were euthanized for the experiments (**Figure 1B**). To characterize the generational effect of DRF on physiology and behaviors, we analyzed three generations (F2, F5 and F10) of DRF mice and *ad libitum* mice. We found that birth weight, litter size and sperm counts were similar among *ad libitum* and DRF groups (**Figure 1C**). Body weight gain in both the DRF and *ad libitum* groups gradually increased from 1 to 2 months of postnatal age (**Figure 1D**). The mice fed with DRF weighed less than the mice fed *ad libitum*, but there was no difference among the DRF groups (**Figure 1D**). The DRF groups ate less food than the *ad libitum* group, but there was no significant difference among generations (**Figure 1E**). Plasma and hepatic triglyceride (TG) levels were comparable among *ad libitum* and DRF groups (**Figure S1A**). Then, to determine whether DRF altered the diurnal rhythms of feeding and other behavior or physiological outputs, we recorded foraging behavior and energy expenditure parameters of the DRF and *ad libitum*-fed mice using the Comprehensive Lab Animal Monitoring System (CLAMS, IC). The mice with imposed DRF shifted their feeding behavior to match the external food accessibility with an enhanced feeding peak in the antiphase to the *ad libitum*-fed mice (**Figure 1F**). The activity pattern was disrupted by DRF, which induced additional activity during food accessibility time (**Figure 1G**). Consistent with the results of feeding behavior, metabolic parameters, including heat production, oxygen consumption (VO_2_), carbon dioxide production (VCO_2_) and respiratory exchange ratio (RER), exhibited diurnal fluctuation induced by DRF, which was antiphase to those of the *ad libitum*-fed mice (**Figures S1B–S1E**). Taken together, these results demonstrate that multigenerational exposure to an inverted feeding regime causes low weight with behavior and metabolic adaptation to DRF. However, the absence of a significant difference among generations indicated that these changes are driven by external feeding time.

### Reprogramming of the rhythmic liver transcriptome in multigenerational DRF

**Figure 2:**
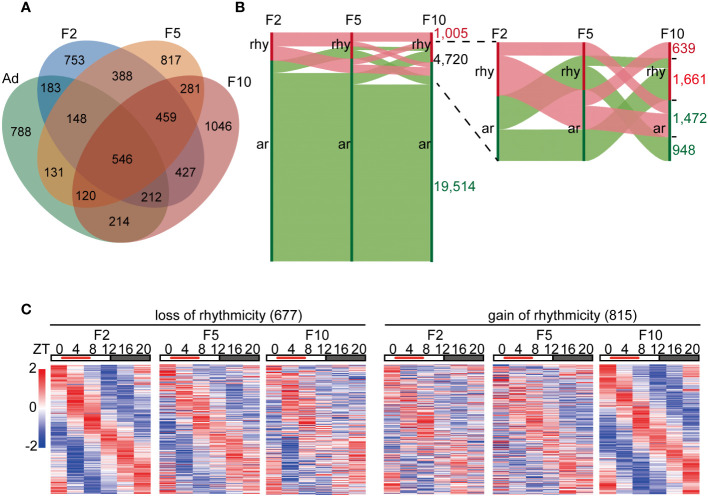
Liver diurnal rhythms are reprogrammed *via* multigenerational DRF. **(A)** Venn diagram displaying the total number of rhythmic genes in the liver from *ad libitum*, F2, F5 and F10, including common genes. **(B)** Changes in gene expression rhythms from F2 to F10. Vertical red bars represent rhythmic genes(rhy), while green bars represent arrhythmic genes(ar). Horizontal red bands represent changes in rhythmic genes, while green bands represent changes in arrhythmic genes in F2. **(C)** Left: heatmaps display generational rhythm-loss genes satisfying the following condition: meta2d_*P*<0.05 in F2, meta2d_*P*>0.05 in F10, and meta2d_*P* in F5 is between meta2d_*P* in F2 and F10. Right: heatmaps display generationally rhythm-gain genes satisfying the following condition: meta2d_*P*>0.05 in F2, meta2d_*P*<0.05 in F10, and meta2d_*P* in F5 is between meta2d_*P* in F2 and F10.

To study the reprogramming of rhythmic gene transcription by DRF over generations, we collected the liver tissues of DRF mice in three generations (F2, F5 and F10) every 4 h for 24 h, and the whole liver transcriptome based on RNA-seq was analyzed, followed by the detection of rhythmic transcripts using the meta2d algorithm. We detected robust rhythmic expression in 3,116 liver transcripts in F2, of which 49.5% ceased to be oscillatory in F5 (**Figure 2A**). Similarly, only 1,406 rhythmic genes were shared between F5 and F10 (**Figure 2A**). Next, Sankey diagrams were used to illustrate the dynamics of liver rhythmicity across generations (**Figure 2B**). Of all detected genes, 1,005 maintained rhythmicity in three generations with comparable phase and amplitude (**Figures 2B**; **S2A-S2D**). Notably, the flow diagram showed that 4,720 genes changed their oscillatory statuses by DRF in at least one generation, while 3,133 (66.4%) genes displayed reversed rhythmicity between F2 and F10 (**Figure 2B**). These genes were grouped into two categories (loss and gain of rhythmicity) based on their meta2d_*P* values from the meta2d algorithm, which determines the extent of rhythmicity (**Figure 2B**). Furthermore, we found that the diurnal oscillation of 677 genes was dampened, and 815 genes were gained over generations (**Figure 2C**). Collectively, our data indicated that the oscillatory liver transcriptome was dramatically reprogrammed *via* multigenerational DRF. Generational gain or loss of rhythmicity occurred in more than 30% of reprogrammed genes.

### Clock gene expression is less affected by DRF

Then, we further detected the response of the liver clock to multigenerational DRF. The expression of different core clock genes (*Arntl*, *Dbp*, *Nr1d1*, *Per1*, *Per2*, *Cry1*) was analyzed by the meta2d algorithm to detect their rhythmicity and estimate their phases and relative amplitude (**Figure S3A**). Except for *Per1* in F10, the expression of liver clock genes was approximately 8 h phase-shifted toward the feeding time by DRF, and the shifted expression pattern was maintained from F2 to F10 (**Figures S3A, S3B**). Loss of circadian rhythmicity in *Per1* was observed in F10 (**Figure S3A**). Interestingly, DRF gradually decreased the amplitude of the circadian output gene *Dbp* generation by generation (**Figure S3A**). Moreover, the phases and amplitude of core clock genes were perturbed upon DRF, and the altered expression pattern was maintained over generations.

### Functional enrichment analysis of genes with generational changes in rhythmicity

**Figure 3:**
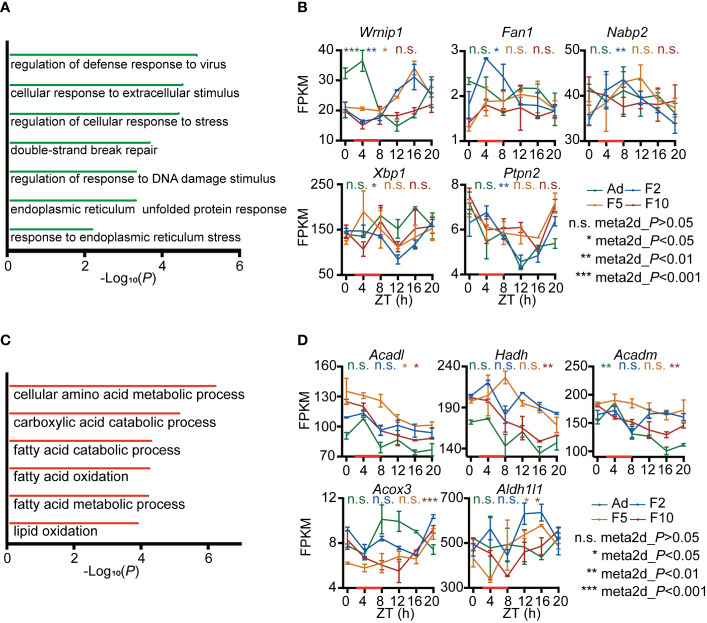
Functional analysis of genes with generational changes in rhythmicity. **(A)** Gene ontology (GO) biological process enrichment analysis of generational rhythm-loss genes during multigenerational DRF. **(B)** RNA-seq expression profiles of representative generational rhythm-loss genes involved in DNA damage and ER stress (n = 2). **(C)** GO biological process enrichment analysis of genes with generational rhythm-gain during multigenerational DRF. **(D)** RNA-seq expression profiles of representative generational rhythm-gain genes involved in fatty acid and lipid metabolism (n = 2). P-values were calculated using the meta2d algorithm; *P < 0.05; **P < 0.01; ***P < 0.001 were considered rhythmic; n.s., not rhythmic. Error bars represent the mean ± SD.

To archive the functional consequences of this reprogramming of rhythmicity in the liver transcriptome, we performed gene ontology (GO) analysis. We observed a significant enrichment for genes that lost rhythmicity gradually from F2 to F10 in GO categories such as response to DNA damage and ER stress (**Figure 3A**). Representative genes, such as *Wrnip1*, *Fan1* and *Nabp2*, which are involved in DNA damage response, exhibited lower expression across 24 h in F10, which resulted in a plateau of expression (**Figure 3B**). *Xbp1* and *Ptpn2*, which are involved in ER stress, fluctuated more in F10 (**Figure 3B**). However, genes that exhibited a generationally gain of rhythmicity in the liver were mainly enriched in fatty acid metabolic processes (**Figure 3C**). DRF in F10 generated harmonic oscillation of *Acadl* (**Figure 3D**). In addition, a deeper trough, but unstable local lowest point, of diurnal expression of *Hadh*, *Acadm*, *Acox3* and *Aldh1l1* was observed in F10 compared to F2 (**Figure 3D**).

### Multigenerational DRF exacerbates ER stress

**Figure 4:**
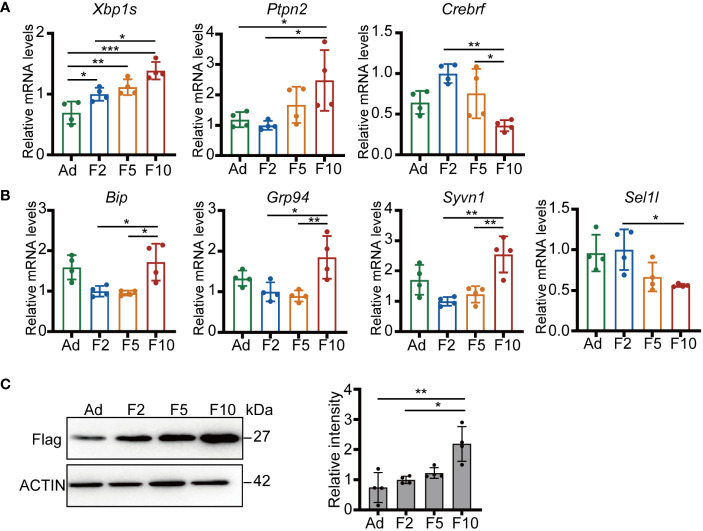
Multigenerational DRF exacerbates ER stress. **(A)** Relative mRNA levels of *Xbp1s*, *Ptpn2* and *Crebrf* (n = 4). **(B)** Relative mRNA levels of *Bip*, *Grp94*, *Syvn1*, and *Sel1l* (n = 4). **(C)** Protein levels of flag-tagged folding-deficient mutant proinsulin 20 h after injection (one representative figure is shown) and quantification of FLAG/ACTIN protein levels (n = 4). All significance was calculated by one-way ANOVA. *P < 0.05; **P < 0.01; ***P < 0.001 were considered significant. Error bars represent the mean ± SD.

To verify that there are indeed differences in the enriched ER stress pathways between generations, we challenged *ad libitum*, F2, F5 and F10 mice with half of the standard dose of the ER stress-inducing agent tunicamycin (TM), which impairs protein folding by blocking N-linked glycosylation (20, 21). First, we examined ER stress-associated gene expression in liver tissues at ZT8 24 h after TM injection. Our data showed that the mRNA levels of *Xbp1* and *Ptpn2* displayed pronounced upregulation in the livers of F10 mice (**Figure 4A**). Conversely, the response of *Crebrf* was attenuated from F2 to F10 (**Figure 4A**). *Xbp1* is a key activated modulator of the UPR, while *Crebrf* participates in the negative regulation of the UPR (22), indicating higher activation of the UPR in the livers of F10 mice. Thus, we next examined other representative genes downstream of the UPR involved in protein folding and degradation. Hepatic transcript levels of the chaperone proteins *Bip* and *Grp94* increased significantly, whereas changes in the critical complex of endoplasmic reticulum-associated degradation (ERAD), an integral part of the UPR (23), were the opposite (**Figure 4B**). Significantly increased transcript levels of the E3 ubiquitin-protein ligase *Syvn1* (*Hrd1*) and decreased transcript levels of the adaptor protein *Sel1l* in the livers of F10 mice were observed (**Figure 4B**).

To further investigate the efficiency of the ERAD machinery, we generated labeled folding-deficient proinsulin in mouse liver by hydrodynamic tail vein injection (24). Intriguingly, more flag-tagged misfolded proinsulin was detected in the livers of F10 mice (**Figure 4C**). Altogether, these results suggested that multigenerational DRF can enhance fundamental processes attempting to restore ER homeostasis and chronically affect the clearance of misfolded proteins.

### Multigenerational DRF induces hepatocyte apoptosis by ER stress

**Figure 5:**
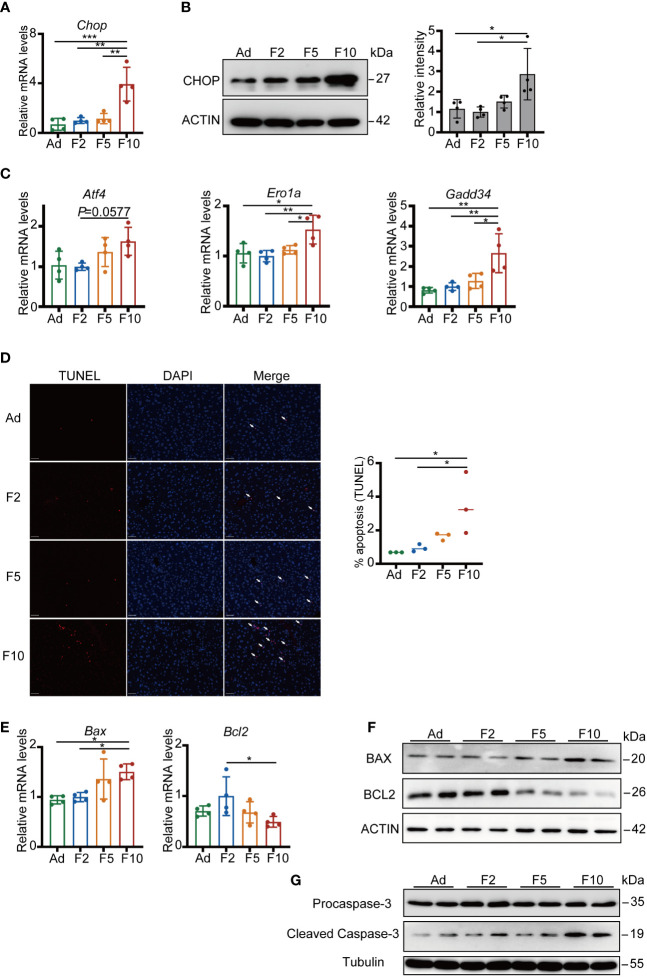
Multigenerational DRF increases ER stress-induced hepatocyte apoptosis. **(A)** Relative mRNA levels of *Chop* (n = 4). **(B)** CHOP protein levels (one representative figure is shown) and quantification of CHOP/ACTIN protein levels (n = 4). **(C)** Relative mRNA levels of *Atf4*, *Ero1a* and *Gadd34* (n = 4). **(D)** Representative TUNEL staining (red) of mouse liver. Nuclei were stained with DAPI (blue); scale bar, 50 μm. Quantification of the percentage of TUNEL-positive cells (n = 3). **(E)** Relative mRNA levels of *Bax* and *Bcl2* (n = 4). **(F)** Protein levels of BAX and BCL2. **(G)** Protein levels of Procaspase-3 and Cleaved Caspase-3. All significance was calculated by one-way ANOVA. *P < 0.05; **P < 0.01; ***P < 0.001 were considered significant. Error bars represent the mean ± SD.

In the presence of unrelieved ER stress, a switch in the UPR from a prosurvival to prodeath phenotype leads to the activation of apoptosis (25). Thus, we examined the response of *Chop* to TM challenge, which is the key apoptosis regulator in ER stress and increased sharply by overwhelming ER stress (26). TM triggered more than 2-fold increases in *Chop* at both the mRNA and protein levels in the livers of F10 mice (**Figures 5A, B**). Furthermore, our results showed that both upstream (*Atf4*) and downstream (*Ero1a* and *Gadd34*) of *Chop* were upregulated in F10 (**Figure 5C**). GADD34 and ERO1A also exert proapoptotic functions downstream of CHOP (27). Thus, we analyzed hepatic apoptosis by terminal transferase-mediated dUTP nick-end labeling (TUNEL) staining. More TUNEL-positive cells were detected in the livers of F10 mice (**Figure 5D**). Moreover, significantly higher mRNA levels of *Bax* and lower levels of *Bcl2* were measured in the livers of F10 mice (**Figure 5E**). Furthermore, the protein levels of BAX and Cleaved Caspase-3 showed an increase, while BCL2 displayed a decreasing tendency in the liver from F2 to F10 (**Figures 5F, G**). These results indicate that CHOP-induced apoptosis was more activated under ER stress stimulation by multigenerational DRF.

Additionally, without TM challenge, the mRNA levels of *Xbp1*, *Ptpn2*, *Crebrf*, *Bip*, *Grp94*, *Syvn1*, *Sel1l*, *Chop*, *Atf4*, *Ero1a*, and *Gadd34* showed similar changes compared to those under stimulation in the livers of F10 mice, suggesting that multigenerational DRF increases basic hepatic ER stress levels (**Figure S4A**). However, few TUNEL-positive cells could be detected, suggesting that ER stress is mild under physiological conditions (**Figure S4B**).

Taken together, our results provide evidence that multigenerational DRF impairs the efficiency of ERAD and activates the CHOP-induced apoptosis pathway under exogenous ER stress stimulation.

## Discussion

Growing evidence emphasizes the importance of eating time, and eating at the wrong time of the day, such as inverted feeding, may have deleterious effects on health. Of particular interest, little attention has been focused on the effect of successive generations of inverted feeding exposure on health. To this end, we developed a mouse model of DRF for 10 successive generations and performed a comprehensive analysis of liver rhythmic transcriptome. By analyzing RNA-seq data from the livers of F2, F5 and F10 mice, we identified two trends of changes in gene rhythmicity, which were dampened or enhanced generation by generation. Genes that lost rhythmicity generationally mainly regulate the response to ER stress, which suggests that accumulated mistimed feeding disrupts protection against environmental stimuli. Indeed, our experimental data from a mouse model of ER stress demonstrated that multigenerational mistimed feeding exacerbates liver injury, as more apoptotic hepatocytes were observed after TM stimulation.

DRF is known to change the phase of the circadian clock and gene expression programs, especially in primary metabolic organs such as the liver. As previously reported, the expression rhythms of most core clock and clock-controlled genes in our research were entrained by restricted feeding (14). Intriguingly, the liver circadian transcriptome was also distinguishable between different generations under the same diet regimen. In addition to epigenetic factors such as histone modification, factors participating in nutrient-sensing pathways may play important roles in multigenerational DRF-induced rhythm reprogramming. The circadian system and nutrient-sensing pathways interact at the whole organism to individual molecule levels. Nutrient-sensing pathways can impact the circadian clock, and conversely, the circadian system also modulates nutrient sensing and response (28). Further research is needed to explore whether nutrient-sensing signals originating from long-term circadian misalignment are disrupted during consecutive DRF.

Interestingly, most liver clock genes kept oscillation during multigenerational DRF except *Per1*, whose rhythmicity was lost in F10. The expression of period genes was acutely affected by refeeding, and the phase change of *Per1* is the most sensitive to external stimuli (29, 30). It is very likely that confliction between antiphase diet and circadian feedback loop might counteract the rhythmicity of *Per1*. ARNTL is one of crucial transcription factors that regulating the expression of *Per1* (31). Although the expression patterns of Arntl were similar among DRF groups, other posttranscriptional regulation may dampen the *Per1* mRNA level. MicroRNAs have been reported to be involved in the posttranscriptional regulation of *Per1* by affecting the stability of mRNA, and microRNA levels are sensitive to nutrition (32–35). Moreover, IRE1α endoribonuclease is one of three main UPR signaling branches and decreases *Per1* mRNA in tumor cells without affecting transcription (36). Our results showed that higher UPR response was detected in the liver of F10 mice. Whether lower mRNA levels of *Per1* at ZT8 were resulted from higher activity of IRE1α endoribonuclease needs to be explored in the future.

In our ER stress model, overwhelming ER stress was observed in the livers of F10 mice upon TM challenge. Although molecular chaperones such as *Bip* and *Grp94* were more highly activated, CHOP-induced hepatocyte apoptosis was increased remarkably. TM induces ER stress in cells by inhibiting the first step in the biosynthesis of N-linked glycans that are necessary for protein folding and maturation. The protein glycosylation process could be impaired by DRF and produce excessive misfolded proteins. The lower efficiency of mutant protein clearance in the livers of F10 mice suggests that the function of the proteasomal degradation system as well as autophagy might be impaired over generations. Thus, multigenerational DRF triggered unbalanced production and clearance of cell debris and resulted in prolonged ER stress.

We have indeed made extensive efforts to phenotyping the effect of DRF on the sperm counts, motility by sperm swimming speed, fertility by *in-vitro* fertilization, mating ability and litter size, but no significant difference was found among generations. Here are some possible factors that mediate the intergenerational effects of multigenerational DRF on offspring. Increasing evidence indicates that non-DNA sequence-based epigenetic information, including DNA methylation, histone modifications and non-coding RNAs, can be inherited across several generations (37). Considering all male and female mice were kept in DRF for life, not only somatic cells but also developing progenitor germ cells might be affected. Thus, epigenetic information both from paternal and maternal may play an important role in offspring during multigenerational DRF. Epigenetic factors modulated by the environmental conditions experienced by parents have the potential to affect the zygote at fertilization, thereby regulate early embryonic development and impact the health of descendants. Although DNA methylation and histone modifications are removed during spermatogenesis and embryogenesis, there are still a minimal fraction of sperm DNA methylation and histone modifications that are preserved and contribute to inter and transgenerational effects on offspring (38–43). Some findings suggest that sperm RNAs functions in the transmission of paternal metabolic disorders associated with diet challenge, thus sperm RNAs may mediate the cumulative effects of multigenerational DRF stimulation probably (44, 45). For maternal factors, DRF exposure exists not only during oogenesis but also during early embryonic development and postnatal lactation. As previously reported, dysfunctional mitochondria in oocyte and abnormal oocyte development have enduring effects on the long-term health of the offspring (46, 47). Furthermore, aberrant nutritional and endocrine levels caused by exposure to adverse environmental stimuli such as undernourishment *in utero* and lactation results in impaired health for progenies (48–50). Thus, the effects of multigenerational DRF on oocytes, intrauterine development and lactation may contribute to the abnormal phenotypes found in F10 mice. Overall, there may be a complex mechanism to explain generational effects caused by multigenerational DRF in our study. Multiple factors including sperm DNA methylation, histone modifications and sperm RNAs, as well as mitochondria in oocytes, intrauterine development and lactation environments are possible carriers of multigenerational inheritance.

In summary, this study is the first to explore the impairment of DRF at long-term and multigenerational levels to the best of our knowledge, which advances our understanding of the importance of eating time. Our results reveal that multigenerational DRF reprograms the liver circadian transcriptome over generations and induces unhealthy alterations in subsequent progenies that are mainly associated with ER stress. The underlying mechanism of this intergenerational process is intriguing and worth exploring in the future, and this process might contribute to not only social-medical but also evolutionary development.

## Data availability statement

The data presented in the study are deposited in the NCBI SRA repository, accession number PRJNA932189.

## Ethics statement

The animal study was reviewed and approved by the Animal Care and Use Committee of the Cambridge-Su Genomic Resource Center, Soochow University (YX-3, YX-2017-2, YX-2021-2).

## Author contributions

KH, ZL, TZ, and YX are responsible for designing research; KH, ZL, WZ, LS, and PY performed research; KH, YG, TZ, and YX performed bioinformatic analysis; KH, TZ, TW, and YX analyzed data; and KH, TZ, and YX wrote the manuscript. All authors contributed to the article and approved the submitted version.
