# Supplementary material for: Multigenerational mistimed feeding drives circadian reprogramming with an impaired unfolded protein response
Source: Front Endocrinol (Lausanne). 2023 Mar 6;14:1157165. doi: 10.3389/fendo.2023.1157165 (PMC10025471; doi:10.3389/fendo.2023.1157165)
Supplement: Supplementary file 1 [file DataSheet_1.docx]

**FIGURE S1**


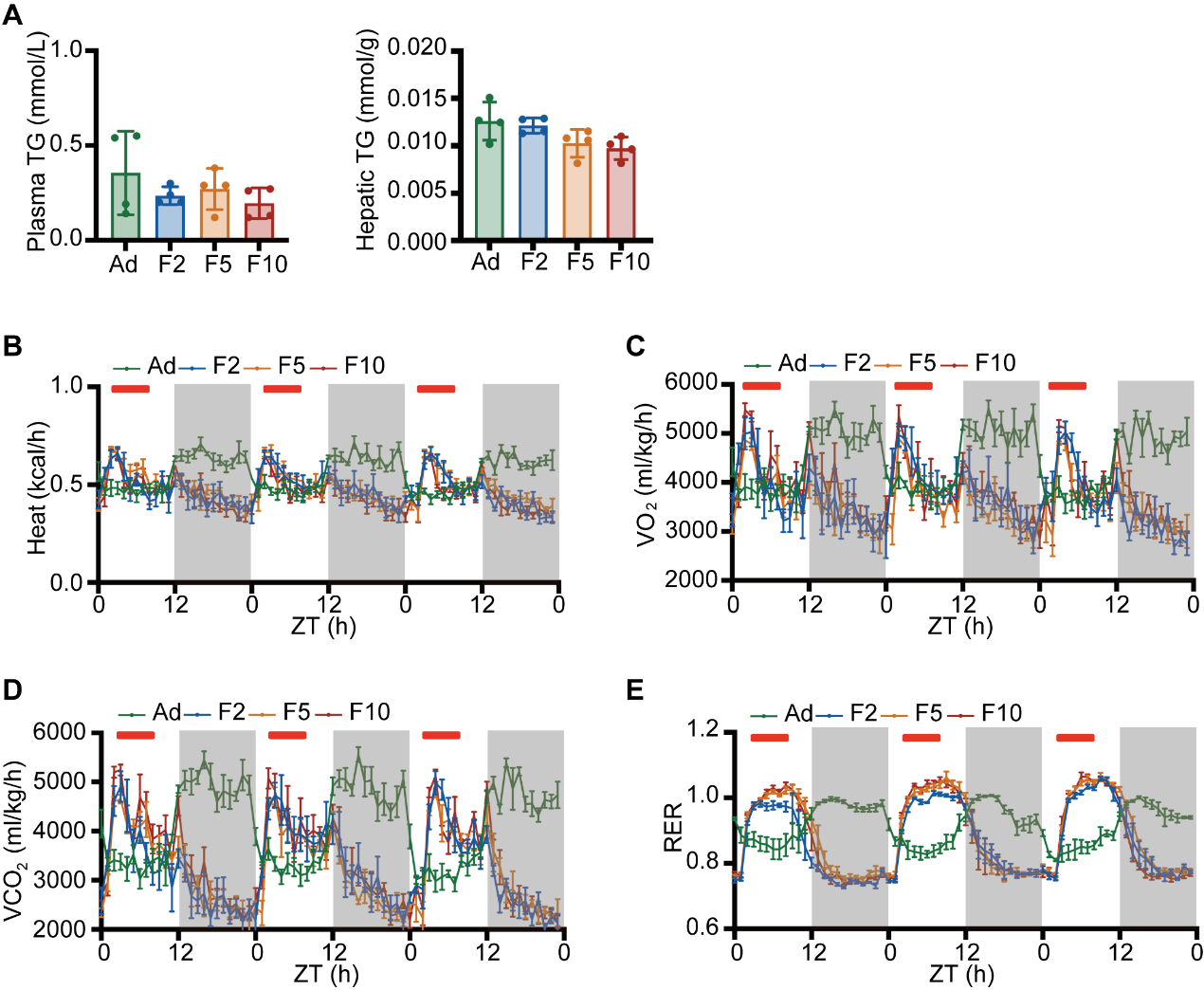


**FIGURE S1|** Basic metabolic measurements (n = 4). Plasma and hepatic triglyceride (TG) levels (**A**), heat production (**B**), oxygen consumption (VO_2_) (**C**), carbon dioxide production (VCO_2_) (**D**) and respiratory exchange ratio (RER) (**E**) of Ad and DRF mice. Red bars indicate timing of food access (Ad group were fed *ad libitum*). Error bars represent the mean ± SD.

**FIGURE S2**


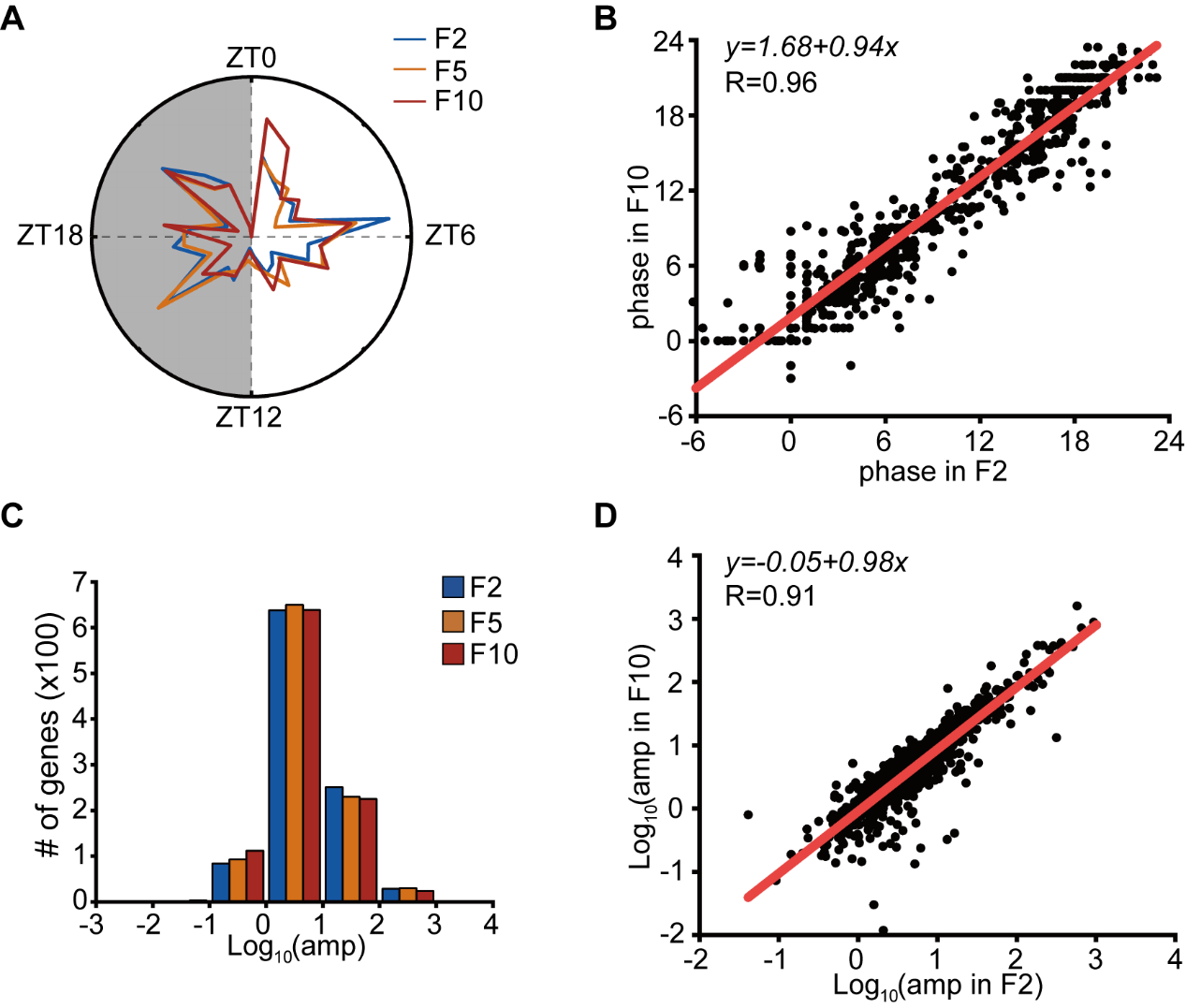


**FIGURE S2|** Common rhythmic genes of DRF mice (n = 2) were screen out through meta2d_*P*. (**A**) Phase distribution of common rhythmic genes in liver of DRF mice. (**B**) Linear regression of phase of common hepatic rhythmic genes from F2 and F10 mice. (**C**) Amplitude distribution of common hepatic rhythmic genes in DRF mice. (**D**) Linear regression of amplitude of common hepatic rhythmic genes from F2 and F10 mice. X and Y-axis denotes Log_10_ amplitude values.

**FIGURE S3**


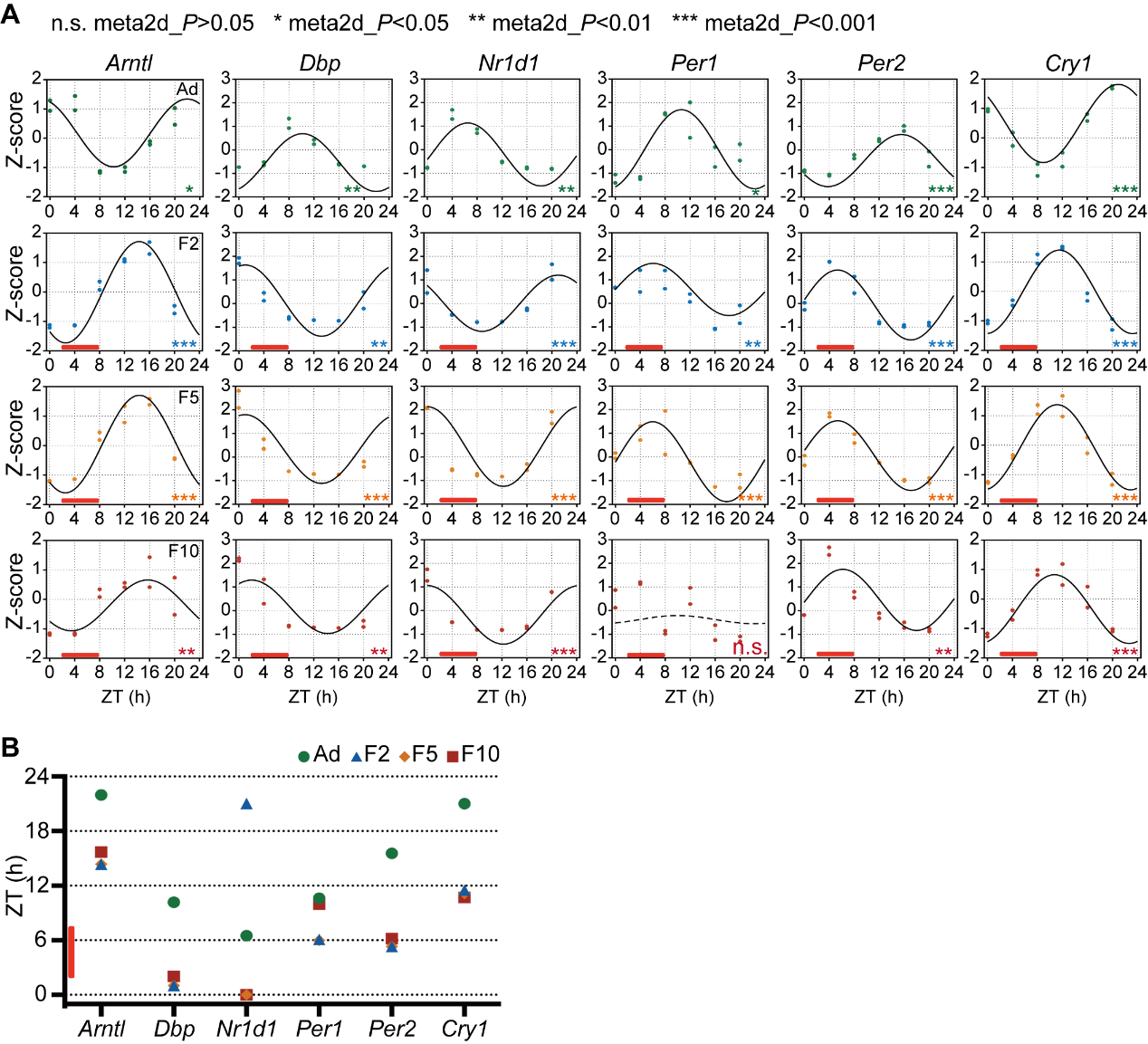


**FIGURE S3|** Multigenerational DRF resets liver clock. RNA-seq results from the Ad and DRF mice, samples were collected at 4 h intervals for 24 h (n = 2 biologically independent mice per time point). **(A)** Presented are clock genes from the liver. Dots mark individual measurements in each Zeitgeber Time (ZT). FPKM values are normalized by Z-score and denoted by cosine fit curve. Red bars indicate timing of food access (Ad group were fed *ad libitum*). **(B)** Phase of clock genes in the livers of Ad and DRF.


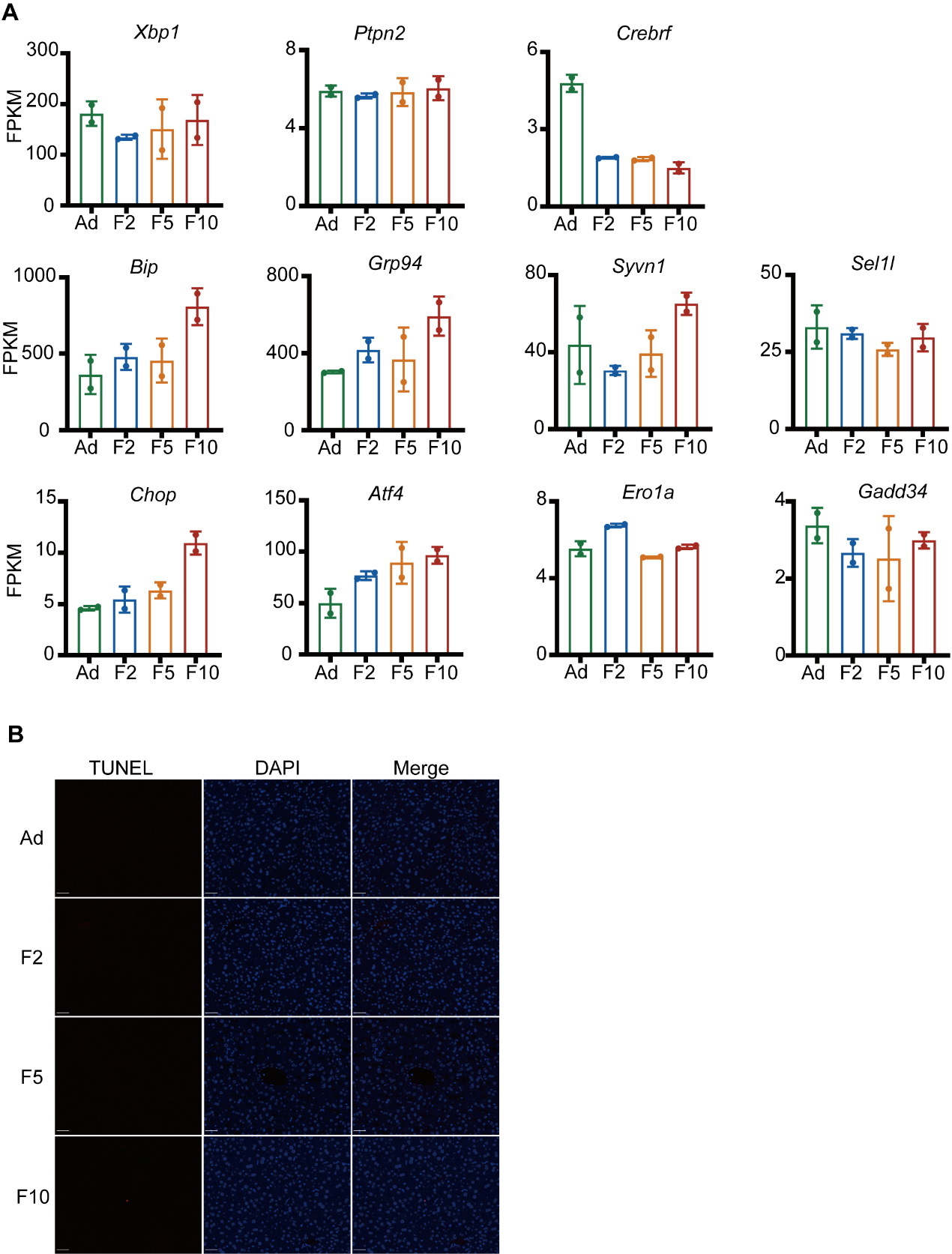
**FIGURE S4**

**FIGURE S4|** **(A)** FPKM levels of ER stress-related genes *Xbp1*, *Ptpn2*, *Crebrf*, *Bip*, *Grp94*, *Syvn1*, *Sel1l*, *Chop*, *Atf4*, *Ero1a* and *Gadd34* without stimulation (n = 2). **(B)** Representative TUNEL staining (red) of mouse liver without stimulation, nuclei were stained with DAPI (blue), scale bar, 50 μm.

**Table S1. Primers for quantitative real-time PCR**

| Genes | Forward primers 5’ to 3’ | Reversed primers 5’ to 3’ |
| --- | --- | --- |
| *Actin* | CTGTCCCTGTATGCCTCTG | ATGTCACGCACGATTTCC |
| *Atf4* | GGGTTCTGTCTTCCACTCCA | AAGCAGCAGAGTCAGGCTTTC |
| *Bax* | AGGATGCGTCCACCAAGAAGCT | TCCGTGTCCACGTCAGCAATCA |
| *Bcl2* | CCTGTGGATGACTGAGTACCTG | AGCCAGGAGAAATCAAACAGAGG |
| *Bip* | GAGGATGTGGGCACGGTGGT | CCCTGATCGTTGGCTATGAT |
| *Chop* | CATACACCACCACACCTGAAAG | CCGTTTCCTAGTTCTTCCTTGC |
| *Crebrf* | CAGCAAGAGAGGATTCTGAGGC | GGTCTTCCACATCAGTTCTCCG |
| *Ero1a* | GGACTGTGTTGGCTGCTTCAAG | GCTGGAACTCATAACTTGGTCCG |
| *Gadd34* | GGCGGCTCAGATTGTTCAAAGC | CCAGACAGCAAGGAAATGGACTG |
| *Grp94* | AAGAATGAAGGAAAAACAGGACAAAA | CAAATGGAGAAGATTCCGCC |
| *Ptpn2* | AAGGTGCAGGATACTGTGGAGG | GCCTCTGTTTCATCTGCTGCAC |
| *Sel1l* | GGAAGTGACATCGTACCTCAGAG | CTTGAACGCCTCTTCCGTAGAG |
| *Syvn1* | CTCATGCCTACTACCTCAAACAC | TGCCCGAAGAACACCTTGC |
| *Xbp1s* | CTGAGTCCGAATCAGGTGCAG | GTCCATGGGAAGATGTTCTGG |
